# Tracking and Monitoring Mood Stability of Patients With Major Depressive Disorder by Machine Learning Models Using Passive Digital Data: Prospective Naturalistic Multicenter Study

**DOI:** 10.2196/24365

**Published:** 2021-03-08

**Authors:** Ran Bai, Le Xiao, Yu Guo, Xuequan Zhu, Nanxi Li, Yashen Wang, Qinqin Chen, Lei Feng, Yinghua Wang, Xiangyi Yu, Chunxue Wang, Yongdong Hu, Zhandong Liu, Haiyong Xie, Gang Wang

**Affiliations:** 1 Advanced Innovation Center for Human Brain Protection Capital Medical University Beijing China; 2 National Engineering Laboratory for Risk Perception and Prevention Beijing China; 3 The National Clinical Research Center for Mental Disorders Beijing Anding Hospital Capital Medical University Beijing China; 4 Beijing University of Posts and Telecommunications Beijing China; 5 Department of Neuropsychiatry and Clinical Neurology Beijing Tiantan Hospital Capital Medical University Beijing China; 6 Department of Psychological Medicine Beijing Chao-Yang Hospital Capital Medical University Beijing China; 7 Department of Neurology, Medical Health Center Beijing Friendship Hospital Capital Medical University Beijing China

**Keywords:** digital phenotype, major depressive disorder, machine learning, mobile phone

## Abstract

**Background:**

Major depressive disorder (MDD) is a common mental illness characterized by persistent sadness and a loss of interest in activities. Using smartphones and wearable devices to monitor the mental condition of patients with MDD has been examined in several studies. However, few studies have used passively collected data to monitor mood changes over time.

**Objective:**

The aim of this study is to examine the feasibility of monitoring mood status and stability of patients with MDD using machine learning models trained by passively collected data, including phone use data, sleep data, and step count data.

**Methods:**

We constructed 950 data samples representing time spans during three consecutive Patient Health Questionnaire-9 assessments. Each data sample was labeled as Steady or Mood Swing, with subgroups Steady-remission, Steady-depressed, Mood Swing-drastic, and Mood Swing-moderate based on patients’ Patient Health Questionnaire-9 scores from three visits. A total of 252 features were extracted, and 4 feature selection models were applied; 6 different combinations of types of data were experimented with using 6 different machine learning models.

**Results:**

A total of 334 participants with MDD were enrolled in this study. The highest average accuracy of classification between Steady and Mood Swing was 76.67% (SD 8.47%) and that of recall was 90.44% (SD 6.93%), with features from all types of data being used. Among the 6 combinations of types of data we experimented with, the overall best combination was using call logs, sleep data, step count data, and heart rate data. The accuracies of predicting between Steady-remission and Mood Swing-drastic, Steady-remission and Mood Swing-moderate, and Steady-depressed and Mood Swing-drastic were over 80%, and the accuracy of predicting between Steady-depressed and Mood Swing-moderate and the overall Steady to Mood Swing classification accuracy were over 75%. Comparing all 6 aforementioned combinations, we found that the overall prediction accuracies between Steady-remission and Mood Swing (drastic and moderate) are better than those between Steady-depressed and Mood Swing (drastic and moderate).

**Conclusions:**

Our proposed method could be used to monitor mood changes in patients with MDD with promising accuracy by using passively collected data, which can be used as a reference by doctors for adjusting treatment plans or for warning patients and their guardians of a relapse.

**Trial Registration:**

Chinese Clinical Trial Registry ChiCTR1900021461; http://www.chictr.org.cn/showprojen.aspx?proj=36173

## Introduction

Depression is a common mental illness characterized by persistent sadness and a loss of interest in activities that people normally enjoy, accompanied by an inability to carry out daily activities for 14 days or longer [1]. The latest estimates from the World Health Organization show that more than 300 million people are now living with depression, and it has increased by more than 18% between 2005 and 2015. Treatment of major depressive disorder (MDD) usually spans a long period (no less than 6 months). Receiving continuous and long-term maintenance treatment could reduce or even prevent relapse. It is essential for doctors to monitor patients’ condition and symptoms to provide appropriate treatment. However, it is impossible for doctors to track the patients’ condition every day as patients revisit their doctors twice a month in an ideal case. Besides, it is not easy for patients to provide a precise description of their conditions for the past several weeks; sometimes, the answer could be as vague as an *OK*.

This study analyzed daily phone usage data, sleep data, and step count data of patients with MDD and their self-evaluated mood scores. According to a study on smartphone ownership across countries, of the top 20 countries reported, an average of 73.45% (SD 10.79%) of adults own a smartphone [2]. According to the China Netcasting Services Association [3], the average time people spend on mobile internet using their smartphones is 341.2 minutes per day in China. With the rapid evolution of smartphone and wearable device technologies, many internet-based mental health services have emerged. Many researchers are focusing on using smartphone usage data to infer mood [4-8]. Sleep and sports data collected by mobile sensors have also been studied by researchers as an inference of mood [9-13]. Jacobson et al [14] used movement and light data to assess depression severity. Cho et al [15] predicted the mood state of patients with MDD in the next 3 days using passively collected data from smartphones. Merikangas et al [16] examined the association among motor activity, energy, mood, and sleep in adults with mental disorders. Cao et al [17] used smartphone-based self-reports, parent evaluations, and passive phone sensor data to monitor depressive symptoms of adolescent patients with MDD. Canzian et al [18] investigated the correlation between patterns of human mobility and emotional states of depressive patients using GPS data collected from smartphones.

When reviewing works on mental state monitoring and predicting, we found that there are 2 major approaches: (1) training a generic model using all data collected and (2) building a personalized model for each patient. During data preprocessing, we observed differences in phone usage routines among patients. Owing to the nature of Patient Health Questionnaire-9 (PHQ-9), which reflects a patient’s mental state for the past week, there were limited data samples for each patient to build a personalized model. To eliminate individual differences between patients, we examined the correlation between the change in phone usage routine, sleep data, and step count and the change in the patient’s level of depression.

The main objective of this study is to examine the feasibility and technical foundation of monitoring variations in depression levels in patients with MDD during a period based on the amount of variation in smartphone usage data, sleep data, and step count data. We then analyzed different models trained by data to determine which types of behaviors were most affected by the change in their depression level.

## Methods

### Smartphone-Based Depression App Design

**Figure 1:**
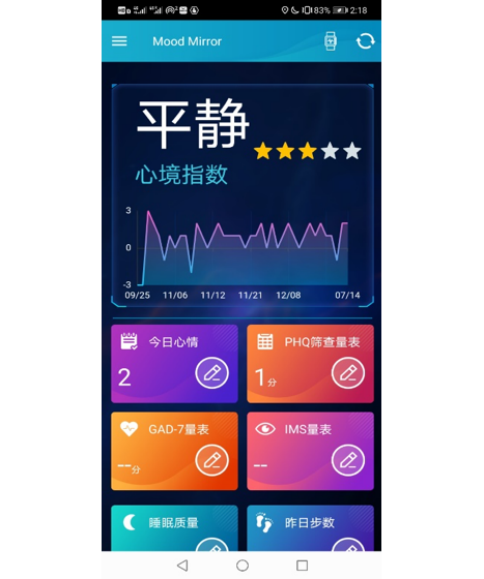
Home screen of the Mood Mirror app.

**Figure 2:**
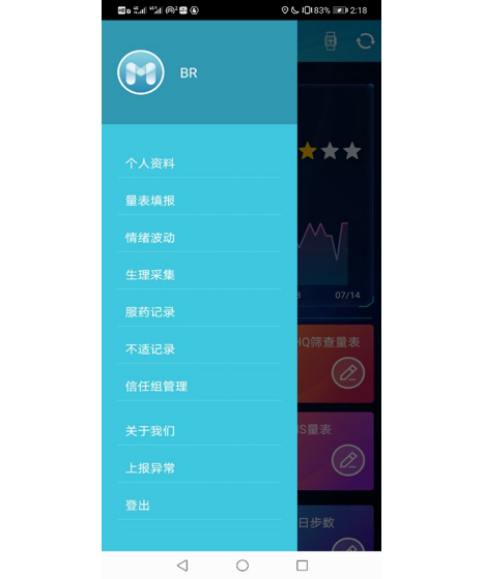
Screenshot of the menu page.

We designed an app called Mood Mirror to track and record patients’ daily activities and mood (Figures 1 and 2). The goal was to collect phone usage data and physical data passively with minimal human action. Owing to the limitations of access to app usage on the iOS platform, our Mood Mirror app only supported the Android platform. The app requires users to wear a wristband that we provided to collect sleep, heart rate, and step count data.

**Figure 3:**
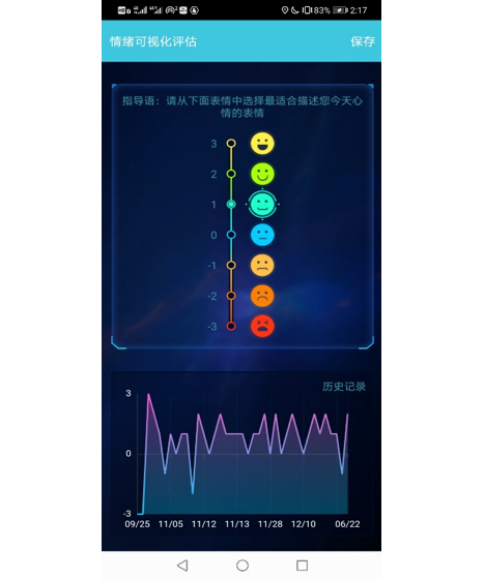
Screenshot of filling the Visual Analog Scale.

**Figure 4:**
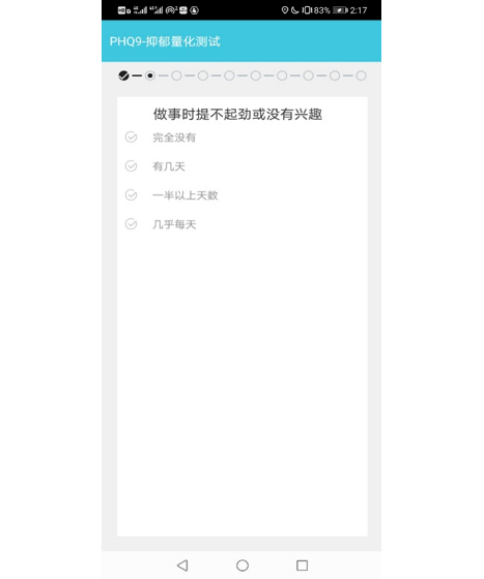
Screenshot of filling Patient Health Questionnaire-9.

The Mood Mirror app consists of 2 main parts: self-evaluation of mood condition and data collection. The app sends a notification to the user every day at 8 PM to use the Visual Analog Scale (VAS) to evaluate their mood of the day on a scale of −3 to 3, with −3 indicating sadness and 3 indicating happiness (Figure 3). The app also provides multiple self-rating tools such as PHQ-9 and Generalized Anxiety Disorder-7 (Figure 4). Users could use these tools to evaluate their mental state anytime. Meanwhile, with users’ consents, the Mood Mirror app runs in the background to collect phone usage data, including call logs, text message logs, app usage logs, GPS, and screen on and off status. These phone usage data would be uploaded instantly to our server. In addition, the app is able to connect with the wristband that is provided via Bluetooth. The data collected by the wristband would first be stored locally and uploaded to our server when the user connects with the wristband using the Mood Mirror app. The Mood Mirror app also allows users to record their medication prescriptions and side effects to keep track of their conditionsAll patients provided written informed consent to participate in the study. Users are able to track their mood variation history, sleep data, and step count via the Mood Mirror app. The Mood Mirror app would send notifications to remind users to keep recording their mood if the app was not used for more than 3 days.

In this study, we selected Mi Band 2 (Xiaomi Corporation), a top-selling wristband model that was sold to millions in China at the time. According to the product description, the data collected were calibrated in their research and development laboratory, and their algorithms of sleep and sports have been widely accepted.

To collect phone usage data that could reflect a subject’s real daily routine, subjects were asked to install the Mood Mirror app on their own phone. The app was tested on more than 20 different models for sale at the time from top-selling brands such as HUAWEI, Xiaomi, and OPPO and had also been tested on different Android operating systems for its compatibility.

### Study Design

This was a multisite, noninterventional prospective study. The study was conducted at 4 psychiatric hospitals or units in general hospitals in Beijing, China. The protocol was approved by the Independent Medical of Ethics Committee Board of Beijing Anding Hospital and the other 3 sites (ethical approval no. 2018-119-201917FS-2). All patients provided written informed consents to participate in the study.

The study was designed to establish a correlation between clinician rating scales, self-rating scales, and passive collected phone usage measures for patients with depression. There were 4 types of data being collected:

1. Physician rating scales, including the Hamilton Depression Rating Scale, were performed by psychiatrists at each visit.
2. Self-rating scales, including PHQ-9, were performed by participants biweekly via the Mood Mirror app.
3. Daily immediate mood was recorded by participants using the VAS via the Mood Mirror app.
4. Phone usage data, including call logs, text message logs, app usage logs, GPS, and screen on and off status, were analyzed.
5. Wristband data, including sleep data, step count, and heart rate, were analyzed.

The study lasted for 12 weeks, and all participants were asked to check in with their doctors and complete the self-rating scales at weeks 0, 2, 4, 8, and 12. There was no restriction to their treatment.

All participants were explained about the study, the design of the app, and the types of data being collected by it. Each participant was then instructed to install the Mood Mirror app on his or her personal smartphone and given a wristband. Participants would connect the wristband to the app and allow the app to gain access to certain data under the assistance of a research assistant and complete self-rating scales.

During the follow-up visits, all participants were asked to record their mood status daily and complete PHQ-9 biweekly via the Mood Mirror app.

### Participants

All participants were recruited from outpatient clinics at 4 sites in Beijing from February 2019 to April 2020. Participants were outpatients aged 18 to 60 years and had been diagnosed with MDD according to *the* *Diagnostic and Statistical Manual of Mental Disorders, fourth edition* criteria. Participants were excluded if they had Axis I primary psychiatric diagnosis other than MDD or had a diagnosis of substance abuse. Clinicians introduced the study to patients who met the study criteria in outpatient clinics. If the patients who own an Android phone were interested, the clinician would refer the patients to the research center, and a research assistant would explain the study in detail. If the patients agreed to participate in the study, the research assistant would ask them to sign an informed consent form and help with the app and wristband setups. Participants received ¥100 (US $15.5) for each follow-up visit.

### Data Preprocessing and Feature Extraction

#### Data Preprocessing

The focus of this study is to monitor mood changes in patients with depression. To do so, the data needed to be resampled and labeled.

**Figure 5:**
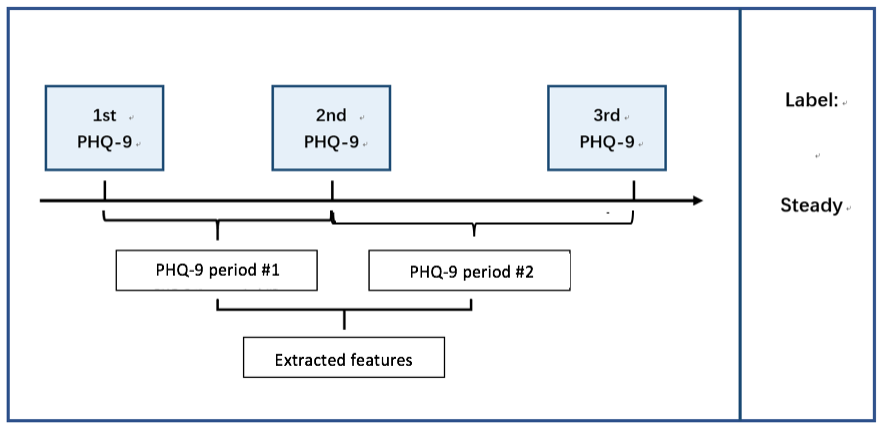
Example of forming a data sample. PHQ-9: Patient Health Questionnaire-9.

For each patient, every 3 consecutive PHQ-9 results and the data collected between the first and the last PHQ-9 evaluation day would be treated as 1 data sample. The data were then divided into 2 parts: (1) data collected between the first and second PHQ-9 evaluation day and (2) data collected between the second and third PHQ-9 evaluation day. These 2 parts are called PHQ-9 periods (Figure 5). As participants were allowed to complete the PHQ-9 tests and submit the scores at any time, the sample would be discarded if either period lasts less than 1 week as the PHQ-9 test mostly reflects the patient’s mental state for the past week. The sample would also be discarded if there were less than 3 days of effective data in either period. On the basis of this standard, the compliance rates for phone usage, call logs, and wristband data are 65.3%, 71.1%, and 58.11%, respectively.

The samples were then labeled into 2 groups and 4 subgroups using 3 PHQ-9 results of each data sample according to the criteria shown in Table 1.

**Table 1:** Data labels and criteria.

Label		Criteria
**Steady**		
	Remission	All three PHQ-9^a^ results≤5
	Depressed	All three PHQ-9 results≥11 and PHQ-9_max_−PHQ-9_min_<5
**Swing**		
	Drastic	PHQ-9_max_−PHQ-9_min_≥10
	Moderate	PHQ-9_max_−PHQ-9_min_≥5

^a^PHQ-9: Patient Health Questionnaire-9.

#### Feature Extraction

As data collected by the smartphone and the wristband were in different forms, the features extracted were different. There were, however, certain types of collected data that were not used in the following study based on common sense judgment and the quality of collected data. For example, text message data were not used because of the popularity of the instant messaging app WeChat. People rarely send text messages using SMS, and there was a large amount of junk messages sent by merchants and service providers. The music data were not used as well; owing to technical problems, the names of the songs were mixed with lyrics and it was difficult to clean the data without human involvement. The details of each data type that were used and extracted features are explained next.

##### Call Logs

It is widely believed that phone call is the key feature that reflects one’s status of social life. For each phone call, the type of call (incoming, outgoing, or rejected), duration, and phone number were logged. The time of the call being made (by hour), the duration of each phone call, the number of different people the phone call was made to or from, and the entropy of callers were extracted from each type of call (incoming, outgoing, and rejected) and for all phone calls during each period.

The entropy H(X) was calculated as follows:

H(X)=-Σ P(X)log_2_[P(X)]

where P(X) is the probability of the occurrence of event X.

Each caller was considered as an event, and the probability was calculated based on the number of times he or she called, was called, or was rejected.

The difference, mean value, and SD of each feature from both PHQ-9 periods were then calculated for each data sample.

##### Phone Usage

The overall phone usage was calculated based on the phone screen on and off status. The Mood Mirror app logged the timestamp when the smartphone was activated or locked by the user either automatically or manually. The number of times and the duration of smartphone used were calculated by screen on and off data. The average and median of phone usage duration and the average and median of the number of times of phone usage were calculated for each period. In addition, the average duration of phone usage for each period the phone was activated was calculated. The ratio of the phone usage duration in the morning (6 AM to noon) to all day phone usage duration was calculated as well as the ratio in the afternoon (noon to 6 PM) and the ratio at night (6 PM to midnight).

The difference, mean value, and SD of each feature from both PHQ-9 periods were then calculated for each data sample.

##### App Usage

Apps were grouped into the following 8 categories (Table 2).

For each group, the following features were calculated:

1. The average, SD, and entropy of the app usage duration.
2. The duration of app usage in the following period: midnight to 3 AM, 3 AM to 6 AM, 6 AM to 9 AM, 9 AM to noon, noon to 3 PM, 3 PM to 6 PM, 6 PM to 9 PM, 9 PM to midnight.
3. The average, SD, and entropy of the number of times of apps being used.
4. The number of times apps were used in the following period: midnight to 3 AM, 3 AM to 6 AM, 6 AM to 9 AM, 9 AM to noon, noon to 3 PM, 3 PM to 6 PM, 6 PM to 9 PM, 9 PM to midnight.

The entropy H(X) was calculated as follows:

H(X)=-Σ P(X)log_2_[P(X)]

where P(X) is the probability of the occurrence of event X.

**Table 2:** Apps categories and examples.

Categories	Examples
Instant messaging	WeChat, QQ
Social networking	Weibo, Zhihu, XiaoHongShu
Shopping	Taobao, JD, PinDuoDuo
Entertainment	TikTok, Bilibili, Youku, iQiyi
Music	Netease Music, QQ Music, Xiami Music
Food delivery	Meituan, Ele.me
Others	Baidu browser, Youdao Dictionary
All apps	All apps being used

Each app category was considered as an event, and the probability was calculated based on the number of times and the duration of that category of app being used.

As messaging is one of the most common ways that people are using recently to communicate with each other, the ratio of the duration of using instant messaging apps to the duration of all apps being used was calculated as a feature to partially represent one’s social life.

The difference, mean value, and SD of each feature from 2 PHQ-9 periods were then calculated for each data sample.

##### Sleep and Step Count

The sleep and step count data were collected using a wristband. There are 4 types of wristband data: activity, light sleep, deep sleep, and not worn.

The wristband uploaded one data packet per minute, containing timestamp, data type, activity intensity, step count, and heart rate.

For sleep data, the average, median, and SD of light sleep, deep sleep, and total sleeping durations were calculated. The ratio of the light sleep duration to the total sleep duration and the ratio of the deep sleep duration to the total sleep duration were calculated as a reference of sleep quality. The time of falling into sleep and wake-up time were also used as features to estimate the user’s daily routine.

For step count data, the total step count for each period was calculated. The average, median, and SD of daily step count and of the following period were calculated as well: midnight to 3 AM, 3 AM to 6 AM, 6 AM to 9 AM, 9 AM to noon, noon to 3 PM, 3 PM to 6 PM, 6 PM to 9 PM, and 9 PM to midnight.

The difference, mean value, and SD of each feature from both PHQ-9 periods were then calculated for each data sample.

##### Heart Rate

Heart rate data were collected using a wristband with a sampling rate of one piece of data per minute. Heart rate data were collected only when the wristband detected the user was in a light sleep mode or in a deep sleep mode.

A cosinor analysis (cosine curve fitting) was performed on heart rate data of each night. The amplitude, acrophase (peak), mesor (mean), and r-squared value (strength) were then generated from the cosine curve, and the average, median, and SD were calculated.

The difference, mean value, and SD of each feature from both PHQ-9 periods were then calculated for each data sample.

### Feature Selection and Machine Learning Models

#### Feature Selection

With all calculated features, it was important to determine which subset of features could best describe the difference between participants who were in a steady mood and those with a mood swing. In this study, 2 different feature selection models were experimented to find a better subset of features that delivered the best accuracy and recall of classification and to avoid overfitting of data.

##### L1-Based Feature Selection

The L1-based feature selection method takes advantage of the fact that linear models using L1 regularization have sparse solutions. L1 regularization adds the sum of the absolute values of the coefficient as a penalty term. Owing to the inherent linear dependence on the model parameters, L1 regularization disables irrelevant features and produces sparse sets of features [19].

##### Tree-Based Feature Selection

The tree-based feature selection method adopts the interpretability of the tree model. The importance score of each feature is calculated, with each feature contributing to the final decision. By ranking all the importance scores, the features with lower scores contribute less to the final decision and can be removed.

#### Machine Learning Models

In this study, some of the most classic machine learning (ML) models were deployed to learn from the features extracted earlier and make predictions. To obtain a more accurate result, 10-fold cross-validation was performed for each subset of features of each model.

The average accuracy rate and recall rate of all 10 folds were calculated to estimate the performance of the model.



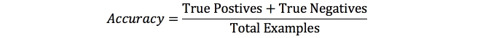





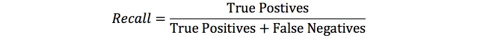



The ML models used were support vector machines (SVMs), K-nearest neighbors, decision trees, naïve Bayes, random forest, and logistic regression.

##### SVM

An SVM is a supervised ML model that can be used for classification. The SVM algorithm creates a line, a hyperplane, or a set of hyperplanes and maximizes the margin around it to separate data into classes.

##### Decision Tree

A decision tree is a tree-like predictive model. In a decision tree, each interior node represents an input feature, the leaf node represents the class label, and the branches represent the decision-making progress from nodes to leaves.

##### Random Forest

**Figure 6:**
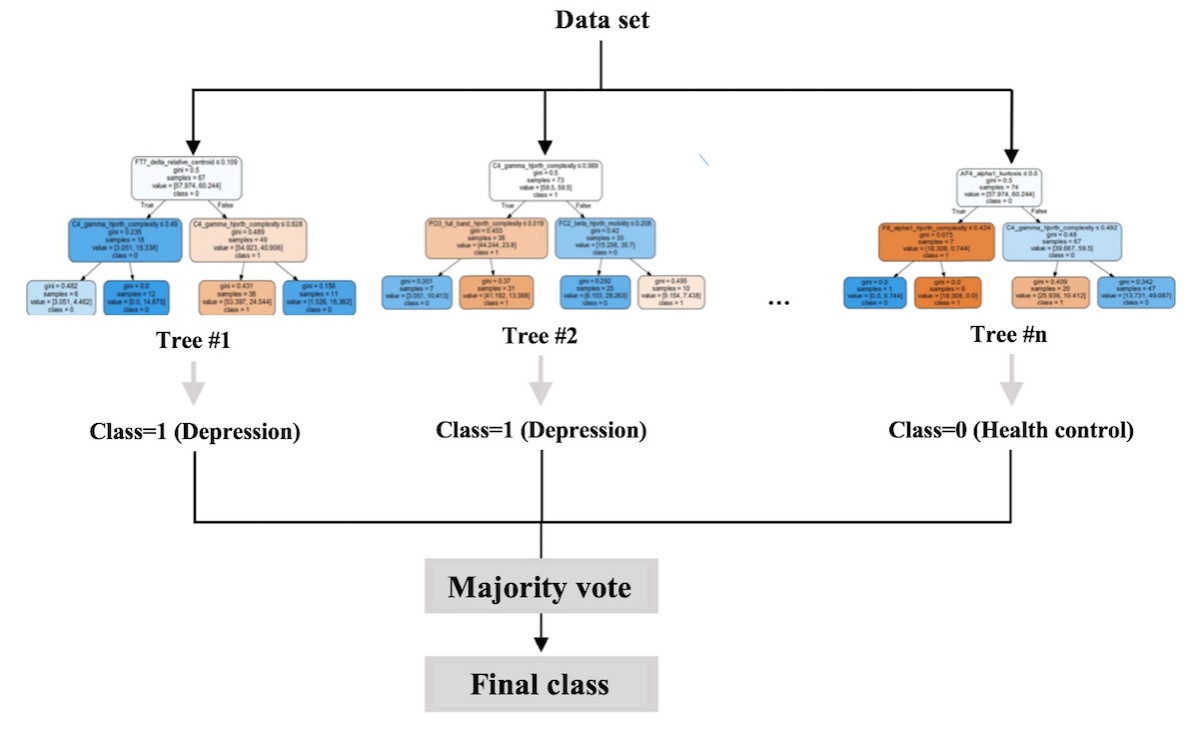
Mechanism of a random forest.

Random forests, as shown in Figure 6, are a combination of tree predictors such that each tree depends on the values of a random vector sampled independently and with the same distribution for all trees in the forest [20]. It is an ensemble learning method for classification. Random forests grow many decision trees. When classifying, the input is put to each decision tree and each tree returns a classification result, and the trees *vote* for the final result. The forest then returns the final classification result with the most votes [21].

## Results

A total of 334 participants were enrolled in this study. Owing to technical limitations and participants’ different degrees of involvement, the amount of usable data samples is limited. Of 334 participants, 261 contributed 950 data samples that were suitable for analysis. As the data collection mechanisms differed between the Mood Mirror app and the wristband, there were discrepancies between the number of phone usage data samples and the number of sleep data samples. The numbers of data samples used for each model are shown in Tables 3-8.

**Table 3:** Classification result using selected features of phone data.

Two classes or subclasses being predicted (number of data samples)	Features selected, n	Best ML^a^ model	Average percent accuracy (SD)	Average percent recall (SD)
Steady (n=144) and Swing (n=234)	4	Random forest	66.76 (4.94)	80.93 (7.72)
Steady-remission (n=25) and Swing-drastic (n=75)	36	Random forest	70.74 (6.62)	77.58 (7.12)
Steady-remission (n=25) and Swing-moderate (n=159)	7	Random forest	80.92 (5.34)	95.50 (2.30)
Steady-depressed (n=119) and Swing-drastic (n=75)	10	Decision Tree	66.18 (6.31)	65.71 (6.99)
Steady-depressed (n=119) and Swing-moderate (n=159)	34	Random forest	75.23 (3.75)	88.99 (6.00)

^a^ML: machine learning.

**Table 4:** Classification result using selected features of sleep data.

Two classes or subclasses being predicted (number of data samples)	Features selected, n	Best ML^a^ model	Average percent accuracy (SD)	Average percent recall (SD)
Steady (n=230) and Swing (n=382)	48	Random forest	72.70 (4.74)	90.80 (3.92)
Steady-remission (n=88) and Swing-drastic (n=124)	44	Random forest	77.34 (7.50)	90.61 (6.23)
Steady-remission (n=88) and Swing-moderate (n=258)	17	Random forest	84.46 (5.94)	97.38 (2.95)
Steady-depressed (n=142) and Swing-drastic (n=124)	48	Random forest	68.87 (9.34)	67.09 (9.19)
Steady-depressed (n=142) and Swing-moderate (n=258)	5	Random forest	74.75 (5.96)	90.37 (5.18)

^a^ML: machine learning.

**Table 5:** Classification result using selected features of step count data.

Two classes or subclasses being predicted (number of data samples)	Features selected, n	Best ML^a^ model	Average percent accuracy (SD)	Average percent recall (SD)
Steady (n=138) and Swing (n=246)	11	Random forest	69.24 (8.54)	86.97 (7.35)
Steady-remission (n=31) and Swing-drastic (n=78)	10	KNN^b^	76.09 (8.49)	96.53 (5.32)
Steady-remission (n=31) and Swing-moderate (n=168)	9	Random forest	85.42 (5.69)	99.41 (1.76)
Steady-depressed (n=107) and Swing-drastic (n=78)	8	Logistic regression	70.35 (8.57)	84.16 (11.82)
Steady-depressed (n=107) and Swing-moderate (n=168)	12	Random forest	72.33 (7.55)	84.57 (8.41)

^a^ML: machine learning.

^b^KNN: K-nearest neighbors.

**Table 6:** Classification result using selected features of heart rate data.

Two classes or subclasses being predicted (number of data samples)	Features selected, n	Best ML^a^ model	Average percent accuracy (SD)	Average percent recall (SD)
Steady (n=80) and Swing (n=122)	20	Random forest	75.19 (8.38)	91.92 (6.71)
Steady-remission (n=18) and Swing-drastic (n=48)	9	KNN^b^	75.48 (16.53)	85.17 (15.10)
Steady-remission (n=18) and Swing-moderate (n=74)	13	KNN	82.67 (10.03)	97.64 (4.73)
Steady-depressed (n=62) and Swing-drastic (n=48)	8	Decision tree	74.55 (13.97)	73.79 (16.04)
Steady-depressed (n=62) and Swing-moderate (n=74)	18	Random forest	69.29 (13.21)	75.16 (13.96)

^a^ML: machine learning.

^b^KNN: K-nearest neighbors.

**Table 7:** Classification result using selected features of all data collected.

Two classes or subclasses being predicted (number of data samples)	Features selected, n	Best ML^a^ model	Average percent accuracy (SD)	Average percent recall (SD)
Steady (n=79) and Swing (n=122)	75	KNN^b^	76.67 (8.47)	90.44 (6.93)
Steady-remission (n=18) and Swing-drastic (n=48)	7	Naïve Bayes	74.29 (9.27)	84.31 (10.89)
Steady-remission (n=18) and Swing-moderate (n=74)	8	KNN	80.56 (15.28)	97.08 (5.91)
Steady-depressed (n=61) and Swing-drastic (n=48)	7	Logistic regression	75.91 (13.18)	89.83 (10.34)
Steady-depressed (n=61) and Swing-moderate (n=74)	12	SVM^c^	74.73 (8.44)	83.95 (12.27)

^a^ML: machine learning.

^b^KNN: K-nearest neighbors.

^c^SVM: support vector machine.

**Table 8:** Classification result using selected features of call logs, sleep data, step count data, and heart rate data.

Two classes or subclasses being predicted (number of data samples)	Features selected, n	Best ML^a^ model	Average percent accuracy (SD)	Average percent recall (SD)
Steady (n=79) and Swing (n=122)	37	Random forest	75.64 (5.09)	89.93 (7.26)
Steady-remission (n=18) and Swing-drastic (n=48)	8	Naïve Bayes	81.67 (15.32)	93.33 (10.41)
Steady-remission (n=18) and Swing-moderate (n=74)	7	Decision tree	80.56 (10.49)	92.88 (10.43)
Steady-depressed (n=61) and Swing-drastic (n=48)	35	Random forest	84.27 (14.36)	85.33 (15.72)
Steady-depressed (n=61) and Swing-moderate (n=74)	25	SVM^b^	77.86 (8.90)	88.99 (9.76)

^a^ML: machine learning.

^b^SVM: support vector machine.

Table 3 presents the classification results of predicting mood changes using selected features of phone data, including app usage data and call logs. It is observed that the classification between Steady-remission and Swing-moderate has the highest accuracy rate of 80.92% and recall rate of 95.50%. The classification between Steady-depressed and Swing-drastic has the lowest accuracy rate of 66.18% and recall rate of 65.71%. The classification between all Steady status samples and all Swing data samples has an accuracy rate of 66.76% and a recall rate of 80.93%.

Table 4 describes the classification results of predicting mood changes using the selected features of sleep data. The classification between Steady-remission and Swing-moderate has the highest accuracy rate (84.46%) and recall rate (97.38%). The classification between Steady-depressed and Swing-drastic has the lowest accuracy rate of 68.87% and recall rate of 67.09%. The classification between all Steady data samples and all Swing data samples has an accuracy rate of 72.70% and a recall rate of 90.80%.

The classification results of predicting mood changes using selected features of step count data show that the classification between Steady-remission and Swing-moderate has the highest accuracy rate of 85.42% and recall rate of 99.41%. The classification between all Steady data samples and all Swing data samples has the lowest accuracy rate of 69.24% and recall rate of 86.97% (Table 5).

Table 6 presents the classification results of predicting mood changes using the selected features of heart rate data. The classification between Steady-remission and Swing-moderate has the highest accuracy rate of 82.67% and recall rate of 97.64%. The classification between Steady-depressed and Swing-moderate has the lowest accuracy rate of 69.29% and recall rate of 75.16%. The classification between all Steady data samples and all Swing data samples has an accuracy rate of 75.19% and a recall rate of 91.92%.

Table 7 compares the classification results of predicting mood changes using the selected features of all data collected. The classification between Steady-remission and Swing-moderate has the highest accuracy rate of 80.56% and recall rate of 97.08%. The classification between Steady-remission and Swing-drastic has the lowest accuracy rate of 74.29% and recall rate of 84.31%. The classification between all Steady data samples and all Swing data samples has an accuracy rate of 76.67% and a recall rate of 90.44%.

The classification results of predicting mood changes using selected features of call logs, sleep data, step count data, and heart rate data show that the classification between Steady-depressed and Swing-drastic has the highest accuracy rate of 84.27% and recall rate of 85.33%. The classification between all Steady data samples and all Swing data samples has the lowest accuracy rate of 75.64% and a recall rate of 89.93% (Table 8).

## Discussion

### Principal Findings

To our knowledge, this study is the first to investigate the prediction of mood swings in patients with MDD by using the amount of variation in phone data, sleep data, and step count data in a period.

In this study, we calculated over hundreds of features from phone data, sleep data, and step count data and used different feature selection models to find features that could best represent the data. Multiple ML models were applied, and different combinations of types of data were examined to select the types of data to collect for future applications.

Most of the models have accuracies of more than 70%, showing promising results using passively collected phone and wristband data to predict whether patients with MDD have mood swings.

Among the 6 combinations of types of data we experimented, the overall best combination was using call logs, sleep data, step count data, and heart rate data. Accuracies of predicting between Steady-remission and Mood Swing-drastic, Steady-remission and Mood Swing-moderate, and Steady-depressed and Mood Swing-drastic are more than 80%, and accuracies of predicting between Steady-depressed and Mood Swing-moderate and the overall Steady to Mood Swing classification accuracy were over 75%. The features used in this model included the average, SD, and median of the following: sleep duration, deep sleep duration, light sleep duration, the ratio of the deep sleep duration to all-night sleep duration, the ratio of the light sleep duration to all-night sleep duration, step counts for each 3-hour period of a day, number of people called (incoming and outgoing calls), number of rejected calls, number of answered calls, and r-squared of heart rate fitted curves. We consider that the features chosen by the model reflect some of the depressive symptoms (PHQ-9) of patients with MDD: low sleep quality, reduced social interaction, and reduced physical activity. These features are consistent with clinical phenotypes such as sleep disturbance, loss of interest, social isolation, and fatigue.

Comparing all the 6 aforementioned combinations, we found that overall prediction accuracies between Steady-remission and Mood Swing (drastic and moderate) are better than those between Steady-depressed and Mood Swing (drastic and moderate). We think that patients who continuously show depressed symptoms might have a similar behavior pattern to patients who have mood swings. On the other hand, the differences in daily behavior patterns between patients who are in remission and those who have mood swings might be more significant. This could explain why the classification accuracies between all Steady data samples and all Mood Swing data samples are lower, sometimes the lowest among all classifications, even with the largest data training set.

We found that models using features from all collected data had lower accuracies than those using features from all collected data except for app usage data (Tables 7 and 8). This might suggest that the differences in app usage behaviors are insignificant between patients who are in Steady status and those who have mood swings. Meanwhile, among the 6 combinations of types of data, models using phone data, including app usage and call logs, have the lowest overall accuracies.

### Limitations and Future Work

We observed a data imbalance in our data set with a low prevalence of the Steady-remission class. As recruitment was done in the hospital outpatient department, the severity of depressive symptoms among patients was different, and there were limited data samples of patients who were in remission. The imbalance of data caused most of the models mentioned earlier to have a much higher recall rate compared with accuracy rates.

The overall data size was also limited. With a larger data set, the prediction model could be more robust. We recruited 334 participants, and all of them were asked to use the app as frequently as possible to record their mood and depression level for 12 weeks. Owing to certain restrictions on the Android system, it was difficult to keep our app running in the background 24×7 collecting data.

This study has shown the possibility of using digital phenotyping data to detect MDD patients’ mood stability. We are currently working on a new version of the Mood Mirror app; with more utility functions provided and interaction designs, patients could gain more information about their current condition, which could increase patients’ compliance rate and enhance both the size and quality of data. The current prediction model will be installed on this version and will provide predictions of patients’ mood stability. The app would ask for patients’ feedback on the prediction results. The performance of the models could be improved by a larger and more balanced data set along with the prediction results feedback.

### Conclusions

This study verified the feasibility of using the amount of variation in smartphone data, sleep data, and step count data to predict whether a patient with MDD has a mood swing that should be noticed by his or her guardian and doctors. The key novelty of this study is instead of predicting the mood state of a certain point, we focus on the variation of mood over a period using the amount of variation in passive digital data. The study was limited by the imbalance of data samples and the technical constraint that the app only runs on the Android platform.

## Abbreviations

MDD: major depressive disorder
ML: machine learning
PHQ-9: Patient Health Questionnaire-9
SVM: support vector machine
VAS: Visual Analog Scale
