# Correction: Tracking and Monitoring Mood Stability of Patients With Major Depressive Disorder by Machine Learning Models Using Passive Digital Data: Prospective Naturalistic Multicenter Study

**DOI:** 10.2196/30540

**Published:** 2021-06-17

**Authors:** Ran Bai, Le Xiao, Yu Guo, Xuequan Zhu, Nanxi Li, Yashen Wang, Qinqin Chen, Lei Feng, Yinghua Wang, Xiangyi Yu, Chunxue Wang, Yongdong Hu, Zhandong Liu, Haiyong Xie, Gang Wang

**Affiliations:** 1 Advanced Innovation Center for Human Brain Protection Capital Medical University Beijing China; 2 National Engineering Laboratory for Risk Perception and Prevention Beijing China; 3 The National Clinical Research Center for Mental Disorders Beijing Anding Hospital Capital Medical University Beijing China; 4 Beijing University of Posts and Telecommunications Beijing China; 5 Department of Neuropsychiatry and Clinical Neurology Beijing Tiantan Hospital Capital Medical University Beijing China; 6 Department of Psychological Medicine Beijing Chao-Yang Hospital Capital Medical University Beijing China; 7 Department of Neurology, Medical Health Center Beijing Friendship Hospital Capital Medical University Beijing China

In “Tracking and Monitoring Mood Stability of Patients With Major Depressive Disorder by Machine Learning Models Using Passive Digital Data: Prospective Naturalistic Multicenter Study” (JMIR Mhealth Uhealth 2021;9(3):e24365) the authors noted three errors.

1. In the originally published manuscript, there was no equal contribution footnote for authors Ran Bai and Le Xiao. This has been corrected to note that the authors contributed equally to the manuscript.

2. The affiliation for authors Le Xiao, Xuequan Zhu, Nanxi Li, Lei Feng, Gang Wang was incorrectly listed as follows in the original publication:

Beijing Anding Hospital, Capital Medical University, Beijing, China

This has been corrected to the following:

The National Clinical Research Center for Mental Disorders, Beijing Anding Hospital, Capital Medical University, Beijing, China

3. The original order of authors was as follows:

Ran Bai^1,2^, MS; Le Xiao^3^, PhD, MD; Yu Guo^4^, MEng; Xuequan Zhu^3^, MA; Nanxi Li^3^, MD; Yashen Wang^2^, PhD; Qinqin Chen^2^, PhD; Lei Feng^3^, PhD, MD; Yinghua Wang^2^, PhD; Xiangyi Yu^2^, MS; Haiyong Xie^1,2^, PhD; Gang Wang^1,3^, PhD, MD

^1^Advanced Innovation Center for Human Brain Protection, Capital Medical University, Beijing, China

^2^National Engineering Laboratory for Risk Perception and Prevention, Beijing, China

^3^Beijing Anding Hospital, Capital Medical University, Beijing, China

^4^Beijing University of Posts and Telecommunications, Beijing, China

In the corrected version of the manuscript, Chunxue Wang, Yongdong Hu, and Zhandong Liu have been added as coauthors for their contribution to the design of protocol and data collection. All authors agree with the addition and new order of authors. The revised order of authors is as follows:

Ran Bai^1,2*^, MS; Le Xiao^3*^, PhD, MD; Yu Guo^4^, MEng; Xuequan Zhu^3^, MA; Nanxi Li^3^, MD; Yashen Wang^2^, PhD; Qinqin Chen^2^, PhD; Lei Feng^3^, PhD, MD; Yinghua Wang^2^, PhD; Xiangyi Yu^2^, MS; Chunxue Wang^5^, MD, PhD; Yongdong Hu^6^, MD, PhD; Zhandong Liu^7^, MD; Haiyong Xie^1,2^, PhD; Gang Wang^1,3^, PhD, MD

^1^Advanced Innovation Center for Human Brain Protection, Capital Medical University, Beijing, China

^2^National Engineering Laboratory for Risk Perception and Prevention, Beijing, China

^3^The National Clinical Research Center for Mental Disorders, Beijing Anding Hospital, Capital Medical University, Beijing, China

^4^Beijing University of Posts and Telecommunications, Beijing, China

^5^Department of Neuropsychiatry and Clinical Neurology, Beijing Tiantan Hospital, Capital Medical University, Beijing, China

^6^Department of Psychological Medicine, Beijing Chao-Yang Hospital, Capital Medical University, Beijing, China

^7^Department of Neurology, Medical Health Center, Beijing Friendship Hospital, Capital Medical University, Beijing, China

*these authors contributed equally

The correction will appear in the online version of the paper on the JMIR Publications website on June 17, 2021, together with the publication of this correction notice. Because this was made after submission to PubMed, PubMed Central, and other full-text repositories, the corrected article has also been resubmitted to those repositories.

